# Paraffin immunofluorescence for detection of immune complexes in renal biopsies: an efficient salvage technique for diagnosis of glomerulonephritis in dogs

**DOI:** 10.1186/s12917-017-1287-x

**Published:** 2017-12-01

**Authors:** Akira Yabuki, Mariko Sawa, Moeko Kohyama, Takeshi Hamamoto, Osamu Yamato

**Affiliations:** Laboratory of Veterinary Clinical Pathology, Joint Faculty of Veterinary Medicine, Kagoshima University, 1-21-24 Korimoto, Kagoshima, 890-0065 Japan

**Keywords:** Dog, Glomerulonephritis, Immune complex, Immunofluorescence, Paraffin section, Renal biopsy

## Abstract

**Background:**

Renal biopsy is an essential tool for the diagnosis of proteinuric kidney diseases in dogs, and evaluation of immune complexes (IC) by immunofluorescence (IF) of frozen sections (IF-F) is required for the diagnosis of IC-mediated glomerulonephritis (ICGN). However, the use of frozen sections from renal biopsies can have limitations. The aim of this study was to develop a reliable IF method using formalin-fixed and paraffin-embedded (FFPE) sections to detect ICs in dog ICGN.

**Methods:**

Renal biopsy specimens were obtained from dogs with protein-losing nephropathies. FFPE sections were prepared, and eight antigen retrieval pretreatment protocols were performed: digestion with trypsin, microwave (MW) heating in citrate buffer (MW-CB; pH 6.0), MW heating in Tris-EDTA buffer (MW-TEB; pH 9.0), as well as combinations of the above, and a non-treated control.

**Results:**

A combination of trypsin for 30 min (Try-30) and MW-TEB; pH 9.0 was the most effective antigen retrieval pretreatment, with clear positive signals for IgG, IgA, IgM, and C3 detected by IF-FFPE. Granular signals, an important diagnostic indicator of ICGN, were clearly observed by both IF-F and IF-FFPE after combined pretreatment with Try-30 and MW-TEB, and IgG, IgA, IgM, and C3 signals were almost completely matched in all samples by IF-F and IF-FFPE.

**Conclusion:**

IF-FFPE with Try-30 and MW-TEB pretreatment is a valuable technique for the diagnosis of renal diseases in dogs. This method could be an efficient tool when standard IF-F cannot be used, or does not provide useful results due to lack of glomeruli in the specimens for IF-F.

## Background

Renal biopsy is an essential tool for the diagnosis of glomerular diseases, particularly proteinuric kidney disease in dogs [1, 2]. The ability to differentiate between immune complex (IC)-mediated glomerulonephritis (ICGN) and non-ICGN is crucial for appropriate therapeutic decisions [3]. Therefore, evaluation of glomerular ICs is an essential component of the pathology of renal biopsy specimens.

Glomerular ICs can be identified by a combination of electron microscopy and immunofluorescence (IF), and this should be routinely performed as part of the diagnosis of glomerular disease in dogs as in humans [1]. However, evaluation of ICs by IF is not always successful, as occasionally, renal biopsy IF specimens contain few or no glomeruli. This problem is most commonly observed in small dog bleeds because core specimens obtained from small kidneys using a needle biopsy device should be short (< 10 mm), and specimens of good quality are preferentially used for light microscopy. IF is generally performed on unfixed, snap-frozen tissues embedded in optimal cutting temperature compound, but this technique is difficult for ordinary veterinary clinicians without sufficient training, and a cryostat is needed to prepare high quality frozen sections. Although Michel’s transport media could be utilized to submit the specimens to a diagnostic laboratory for IF, low preservation of morphology is sometimes observed.

The use of formalin-fixed paraffin-embedded (FFPE) sections for IC detection has limitations as well. FFPE sections are usually used in enzyme-based immunohistochemistry. In human renal biopsies, antigen retrieval by proteolytic methods works well and is effective for detection of immunoglobulin by immunohistochemistry [4, 5]. However, in our experience, by conducting immunohistochemical analyses with proteolytic methods, it is difficult to distinguish the pattern of the granular glomerular basement membrane (GBM) that indicates the presence of ICs in ICGN, from the linear GBM pattern, which is occasionally observed as a nonspecifically stained pattern.

IF on FFPE (IF-FFPE) sections has been explored as an alternative to standard IF-F. The sensitivity and specificity of this method for the detection of ICs could be improved by prior antigen retrieval (AR) using proteolysis [6, 7], heat [8], or a combination of both [6]. However, the efficacy of IF-FFPE for the detection of ICs in dog renal biopsies has not been evaluated. The aim of this study was to develop a reliable IF-FFPE IC detection method for the diagnosis of glomerular diseases in dogs.

## Methods

### Samples

Tissue samples were obtained from the renal biopsies of dogs (*n* = 12) who had been clinicopathologically diagnosed with a protein-losing nephropathy. The experiments in this study were performed in accordance with the Guidelines for Animal Experimentation of Kagoshima University, Japan (VM15020), and the dog owners provided informed consent for renal biopsies used for the definitive diagnosis of the glomerular disease, and for research use after diagnosis. Histopathological diagnosis of renal biopsies was based on light microscopy (using hematoxylin-eosin, Masson’s trichrome, periodic acid-Schiff, Jones’ methenamine silver, and Congo red stains), transmission electron microscopy, and IF-F for IgG, IgA, IgM, and complement C3. The same antibodies at the same dilutions and incubation periods, which are described in the subsequent section, were also used for the IF-F.

### IF-FFPE pretreatments

Eight AR treatment methods were evaluated in four cases that showed positive granular glomerular staining for IgG, IgA, IgM, and C3, by IF-F. The eight AR treatment methods were as follows: 1) no treatment; 2) incubation with 0.1% trypsin (Sigma-Aldrich, St. Louis, MO, USA) for 30 min at 37 °C (Try-30); 3) microwave (MW) heating in a citrate buffer (CB, pH 6.0; MW-CB); 4) MW in a Tris-ethylenediaminetetraacetic acid (EDTA) buffer (TEB, pH 9.0; MW-TEB); 5) MW-CB after Try-30; 6) MW-CB after 15 min trypsin digestion (Try-15); 7) MW-TEB after Try-30; and 8) MW-TEB after Try-15. MW heating was performed using a domestic MW oven at 750 kW (Panasonic, Japan) by pre-warming for 5 min, heating for 10 min, and cooling for 20 min at room temperature (RT). In addition, the order of trypsin digestion and MW heating was switched for some sections.

### IF-FFPE

FFPE sections (3 μm) were prepared. After deparaffinization and rehydration, AR was performed as described above. Then, sections were washed with 10 mM phosphate buffered saline (PBS, pH 7.4), blocked with 0.25% casein (Sigma-Aldrich) in PBS, and incubated with goat polyclonal antibodies against dog IgG, IgA, IgM, and complement C3 (Bethyl Laboratories, Montgomery, TX, USA) for 30 min at RT. Each antibody was diluted to 1:1000 in blocking solution. After incubation with primary antibodies, the sections were washed with PBS, then incubated with Alexa Fluor 488-conjugated donkey anti-goat IgG (Life Technologies, Paisley, UK) diluted to 1:1000 for 30 min at RT. Normal goat IgG (Santa Cruz Biotechnology, CA, USA) was used instead of a primary antibody on the negative control section. Stained sections were examined with an Olympus BX-53 fluorescence microscope (Tokyo, Japan).

## Results

**Fig. 1:**
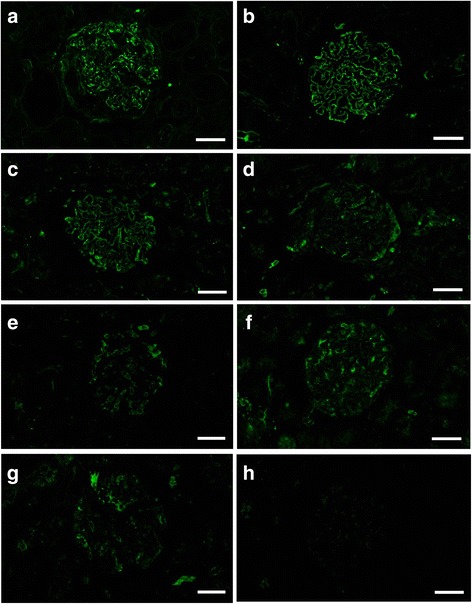
Detection of IgG in a case of ICGN by (**a**) IF-F and (**b**) IF-FFPE after Try-30 and MW-TEB pretreatment. Other AR methods tested in IF-FFPE were as follows: (**c**) Try-15 and MW-TEB, (**d**) Try-30 and MW-CB, (**e**) Try-30, (**f**) MW-TEB, (**g**) MW-CB, and (**h**) no treatment. Each image was obtained from the same case. Scale bars: 20 μm

**Fig. 2:**
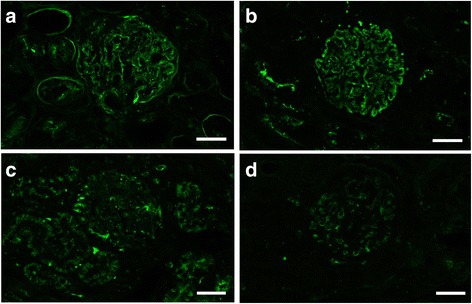
Detection of complement C3 in a case of ICGN by (**a**) IF-F and (**b**) IF-FFPE after Try-30 and MW-TEB pretreatment. **c** IF-FFPE after Try-30 and MW-CB. **d** No treatment. Each image was obtained from the same case. Scale bars: 20 μm

**Fig. 3:**
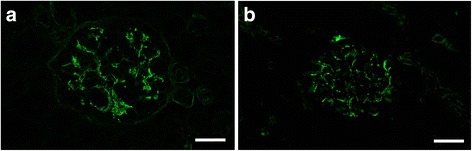
Detection of IgA in a case of ICGN by (**a**) IF-F and (**b**) IF-FFPE after Try-30 and MW-TEB pretreatment. Each image was obtained from the same case. Scale bars: 20 μm

**Fig. 4:**
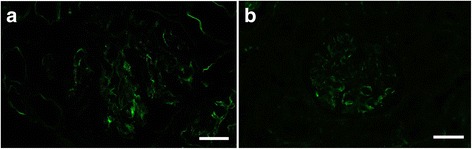
Detection of IgM in a case of ICGN by (**a**) IF-F and (**b**) IF-FFPE after Try-30 and MW-TEB pretreatment. Each image was obtained from the same case. Scale bars: 20 μm

Of the AR pretreatments performed, the best results were obtained from a combination of Try-30 and MW-TEB, with strong positive signals detected for IgG, IgA, IgM, and C3. In cases of ICGN that displayed strong granular signals for IgG and C3 on the glomerular capillary walls by routine IF-F (Figs. 1a and 2a), clear granular signals were also detected in the capillary walls by IF-FFPE (Figs. 1b and 2b). In a case of ICGN that displayed strong granular signals for IgA on the mesangial area of the glomeruli by routine IF-F, clear granular signals for IgA were also detected in the same area by IF-FFPE (Fig. 3). IgM signals by IF-FFPE also matched those observed after routine IF-F (Fig. 4). The IgG, IgA, IgM, and C3 signal intensities in IF-FFPE were strong, and non-specific background staining was not apparent as well as that in IF-F. When the trypsin incubation time was shortened to 15 min (Try-15 with MW-TEB), positive granular signals were still detected in the glomeruli of ICGN cases (Fig. 1c); however, the staining intensities with all antibodies were weaker and the non-specific background signals were stronger compared to Try-30 with MW-TEB. If MW-TEB was performed before Try-30 instead of afterwards, the signals disappeared.

Other tested AR methods did not result in specific granular signals on the glomeruli. When the MW buffer was changed from TEB to CB (Try-30 with MW-CB), granular signals were remarkably weak, and non-granular background signals were seen in the glomeruli and tubules (Figs 1d and 2c). Moreover, the IF-FFPE observations were completely different from routine IF-F. When trypsin incubation alone was performed as a pretreatment (Try-30), strong non-specific signals were observed in the glomeruli and tubules, and granular signals seen in routine IF-F could not be observed by IF-FFPE (Fig. 1e). MW pretreatment alone (MW-CB and MW-TEB) provided similar findings (Figs. 1f and g). IF-FFPE without AR pretreatment displayed only weak non-specific signals (Figs. 1h and 2d).

IF-FFPE with Try-30 and MW-TEB pretreatment was tested using 12 biopsy samples from cases with protein-losing nephropathies (Table 1). In ICGN cases, positive immunoglobulin and C3 patterns by IF-FFPE were almost completely matched to routine IF-F patterns. Findings from cases with non-ICGN and amyloidosis were also quite consistent between IF-FFPE and IF-F, although there were some minor differences (IgG in cases 9 and 10; IgA, IgM, and C3 in case 12).

**Table 1:** Comparison of standard IF-F and IF-FFPE after Try-30/MW-TEB pretreatment

		IgG		IgA		IgM		C3		TEM
Case	Diagnosis	IF-FFPE	IF-F	IF-FFPE	IF-F	IF-FFPE	IF-F	IF-FFPE	IF-F	Dens bodies
1	ICGN	+++	+++^a^	+	+	+	+	+++	++	+++^b^/sepi
2	ICGN	+++	+++	+	+	+	−/+	++	+++	+++/sepi
3	ICGN	++	+	−/+	+	−/+	+	++	++	++/sepi
4	ICGN	+	+	–	–	+	+	+	+	+/pme and sendo
5	ICGN	−/+	−/+	+	−/+	++	++	−/+	+	+/pme and sendo
6	ICGN	−/+	−/+	–	−/+	−/+	+	−/+	+	+/pme and sendo
7	ICGN	−/+	–	+++	+++	+	+	+	+	+/me
8	ICGN	+	+	+++	+++	–	−/+	+	+	+/me
9	Non-ICGN	−/+	–	−/+	−/+	+	−/+	–	−/+	–
10	Non-ICGN	−/+	–	–	−/+	+	−/+	−/+	−/+	–
11	Non-ICGN	–	–	–	–	–	–	–	–	–
12	Amyloidosis	–	–	−/+	–	−/+	–	−/+	–	–

^a^: severity of granular signals in glomeruli; ^b^: severity of dens bodies. *TEM*: transmission electron microscopy, *sepi* subepithelial areas in the glomerular capillaries, *pme* paramesangial areas in the glomeruli, *sendo* subendothelial areas in the glomerular capillaries. *me* mesangial areas in the glomeruli

## Discussion

This study demonstrates that IF-FFPE can be successfully used to detect ICs in renal biopsy specimens from dogs, provided the appropriate AR method is applied before immunostaining. Eight AR methods were tested in this study, and a combination of Try-30 and MW-TEB was the best method for IF-FFPE detection of immunoglobulins and C3 in dog renal biopsy specimens.

The efficacy of IF-FFPE in renal biopsies has been studied in human medicine. In one study, three methods of AR (heating with a Tris buffer of unknown pH, heating with CB, and pronase digestion) were tested, and it was demonstrated that heating with Tris buffer was most effective, allowing the evaluation of ICs in renal biopsy specimens [9]. The efficacy of dual MW-heating methods using an EDTA buffer (pH 8.0) has also been previously reported [8]. In this study, MW heating using a routine protocol (5 min pre-warming, 10 min MW, and 20 min cooling) was tested using CB (pH 6.0) as a standard AR solution and TEB (pH 9.0) as a high pH AR solution. Using the standard IF-F method, specific signals for ICs in ICGN are generally observed as granular signals on glomerular capillary walls or mesangial areas. However, such granular signals were not detected in IF-FFPE with MW heating alone, regardless of the buffer.

Proteinase digestion has also been reported as an effective AR method for IF-FFPE detection of ICs in human renal biopsy specimens [6, 7, 10]. The proteinases examined included trypsin [6], pronase [7], and proteinase K [10], and trypsin digestion was reported to provide successful IF-FFPE detection of immunoglobulins [6]. However, in this study, trypsin digestion alone failed to detect the specific granular signals of ICs, and strong non-specific staining was observed.

A combination of trypsin digestion and MW-heating successfully detected IC immunosignals by IF-FFPE. However, the type of buffer in MW heating affected the specificity and sensitivity, as TEB was suitable, while CB was not, even when combined with trypsin digestion. This difference may be because of the pH of buffers. Trypsin incubation time also influenced the specificity and sensitivity of the immunosignals, with stronger, more specific signals obtained after Try-30 than after Try-15, when combined with MW-TEB. The order of trypsin digestion and MW-TEB was also important, and trypsin digestion should be performed prior to MW-heating. When this order was switched, glomeruli on the sections may have been excessively digested, causing the immunosignals to disappear even if the glomeruli actually contained ICs. This false negative reaction might cause misdiagnosis by failing to distinguish between ICGN and non-ICGN.

Using human renal biopsies, routine IF-F and IF-FFPE have been compared in several reports [7, 10–12]. Although one report suggested that IF-FFPE could not replace IF-F in renal biopsy assessment [12], most studies demonstrated good agreement between the two methods [7, 9–11]. In this study, 12 biopsy samples were used to compare IF-FFPE after Try-30 and MW-TEB pretreatment to routine IF-F. The IF-FFPE observations were almost completely matched to those of IF-F, demonstrating that IF-FFPE with Try-30 and MW-TEB pretreatment could be effectively used for the assessment of ICs in dog renal biopsy specimens. In human renal biopsies, some reports have noted that IF-FFPE is less sensitive than IF-F for the detection of C3 [7, 11]; in this study, this was observed in only the two cases of ICGN with mild IC deposition (Table 1). If a negative reaction for C3 is observed together with positive granular signals for immunoglobulins, diagnosis should be performed carefully in combination with observations by electron microscopy.

In four cases (one case of ICGN, two cases of non-ICGN, and one case of amyloidosis), a small quantity of granular signal was detected by IF-FFPE with Try-30 and MW-TEB pretreatment, while routine IF-F showed no granular signal. It is difficult to determine whether such sparse signals, observed only by IF-FFPE, indicate a small quantity of ICs or artificial products. Although the negative control test using normal IgG showed no granular signals on glomeruli in any of the 12 cases, it does not control for the possibility of artificial products being induced by IF-FFPE. Conversely, it might be possible that the sensitivity of IF-FFPE was indeed higher than routine IF-F, and that small numbers of ICs on the glomeruli may be detectable by IF-FFPE only. Higher sensitivity for IF-FFPE than IF-F has been reported in human renal biopsies [7], with IgG and IgG-kappa detected by IF-FFPE on the glomeruli of many cases, which were negative by routine IF-F. It was suggested that IF-FFPE could arrive at the proper diagnosis when false-negative staining was present by IF-F, as these cases displayed dens deposits on the glomeruli by electron microscopy [7]. In the present cases, diagnosis of non-ICGN was based not only on IF-F but also on electron microscopy, and no dens bodies were detected on any glomeruli. In the case of amyloidosis, the presence of abundant amyloid deposits in the glomeruli was confirmed by polarized light microscopy using the Congo red stain and electron microscopy. Therefore, we are unable to conclude that sparse granular glomerular signals observed only by IF-FFPE represent genuine ICs that displayed false-negative staining by routine IF-F. IF-FFPE described herein should be used as a salvage technique, and faint signals generated by IF-FFPE should not be considered as strong evidence for the presence of ICs. Therefore, if IF-F does not work properly, and IF-FFPE detects faint granular glomerular signals, a definitive diagnosis should be made based on the light and electron microscopic observations.

## Conclusion

In conclusion, IF-FFPE with Try-30 and MW-TEB pretreatment is a valuable technique for the diagnosis of renal diseases in dogs. This technique can clearly detect granular signals indicating the presence of ICs, whose identification by the immunoperoxidase staining of FFPE is difficult. However, if only a small quantity of granular signal is detected on the glomeruli, careful interpretation is required, and diagnosis should be performed in combination with electron microscopy. Although caution is needed when interpreting IF-FFPE results, this method could be an efficient tool when standard IF-F cannot be used, or does not provide useful results due to lack of glomeruli in the specimens.

## Abbreviations

AR: Antigen retrieval (AR)
CB: Citrate buffer
EDTA: Tris-ethylenediaminetetraacetic acid
FFPE: Formalin-fixed paraffin-embedded
IC: Immune complex
ICGN: Immune complex-mediated glomerulonephritis
IF: Immunofluorescence
MW: Microwave
RT: Room temperature
TEB: Tris-ethylenediaminetetraacetic acid buffer
Try-15: 15 min trypsin digestion
Try-30: 30 min trypsin digestion
